# Supplementary material for: Atorvastatin alters the expression of genes related to bile acid metabolism and circadian clock in livers of mice
Source: PeerJ. 2017 May 18;5:e3348. doi: 10.7717/peerj.3348 (PMC5438592; doi:10.7717/peerj.3348)
Supplement: Supplemental Information 1 — Histology photos for ATO [file peerj-05-3348-s002.pptx]

## Slide 1
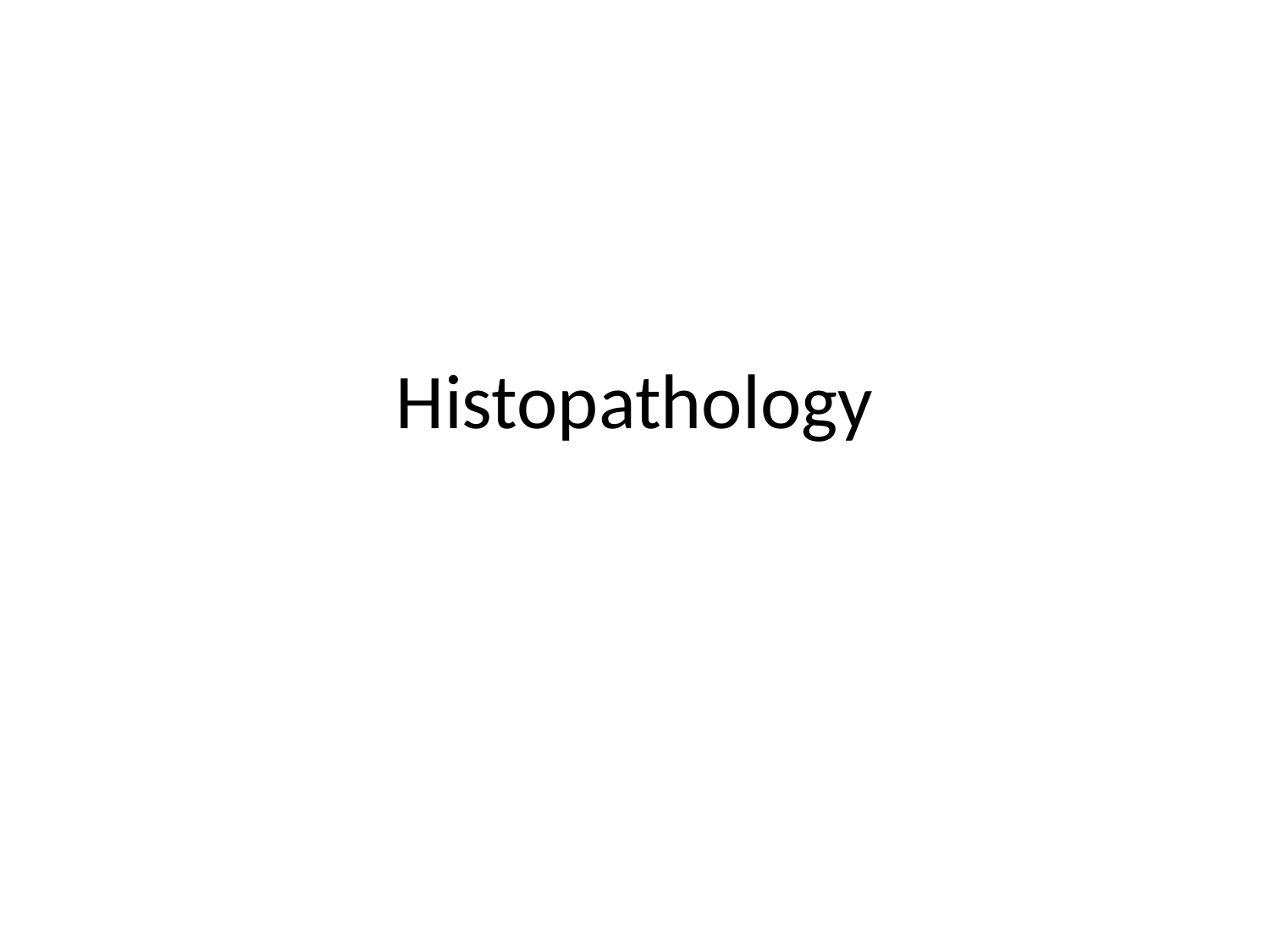

# Histopathology

## Slide 2
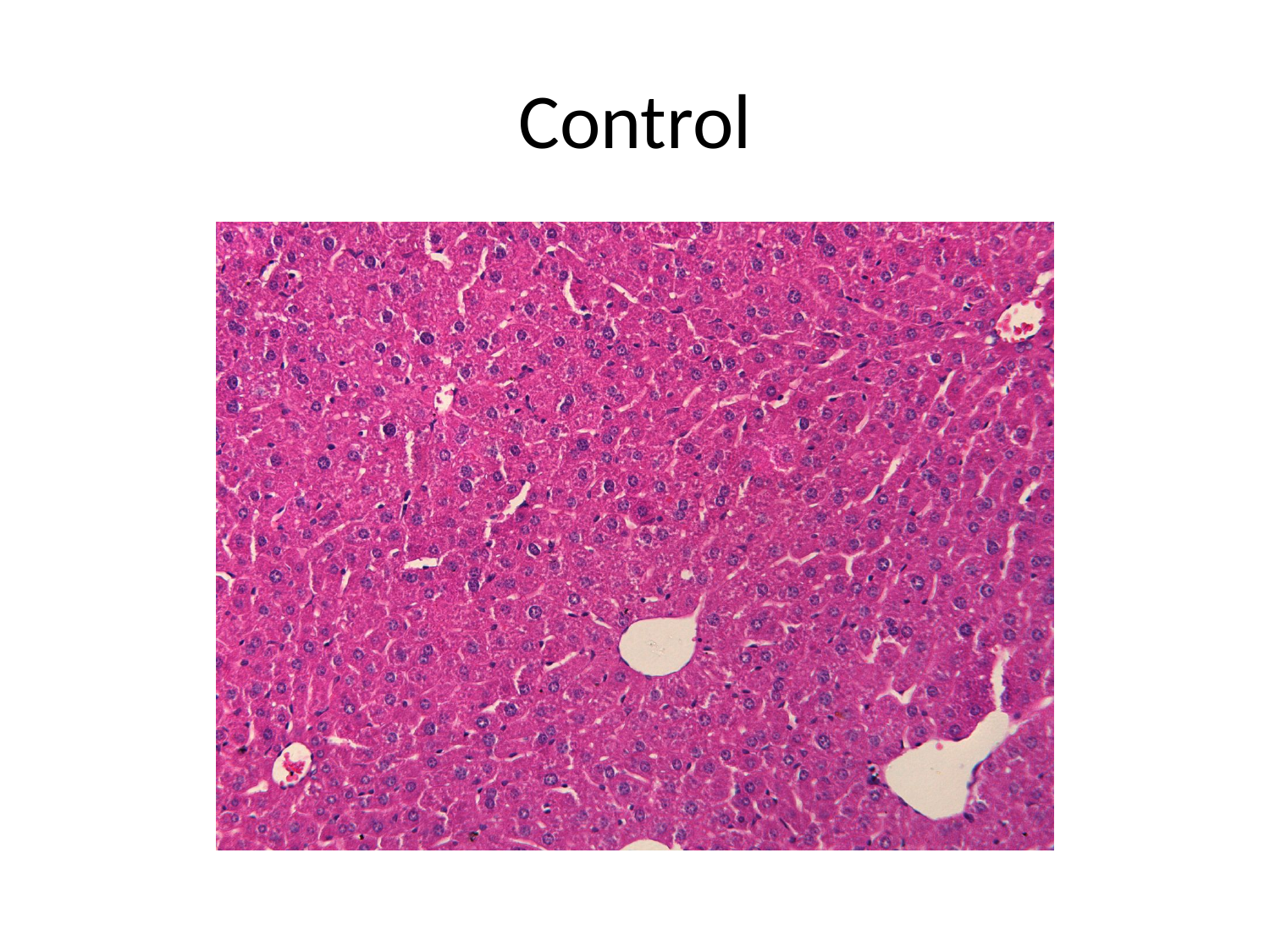

# Control

## Slide 3
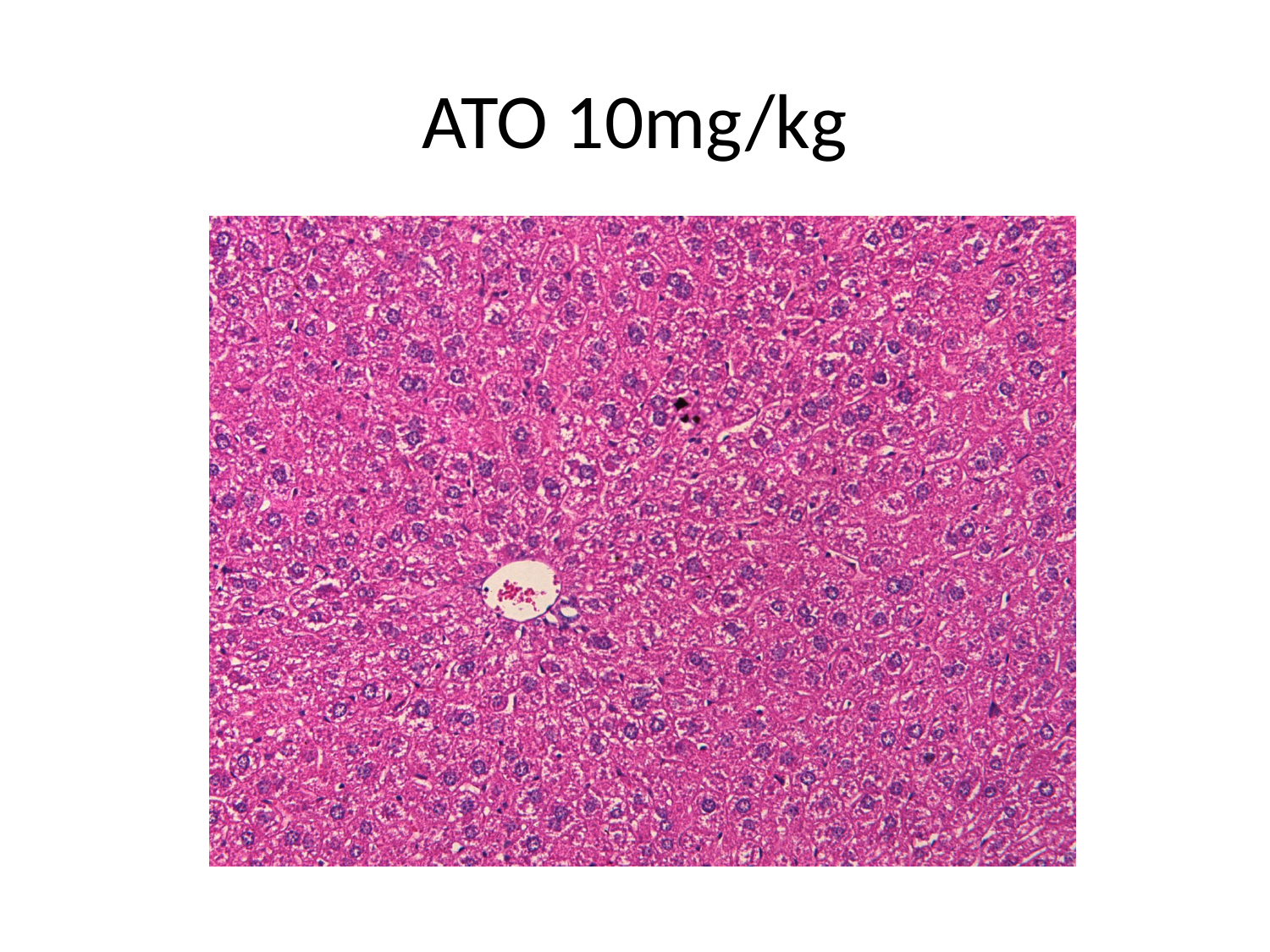

# ATO 10mg/kg

## Slide 4
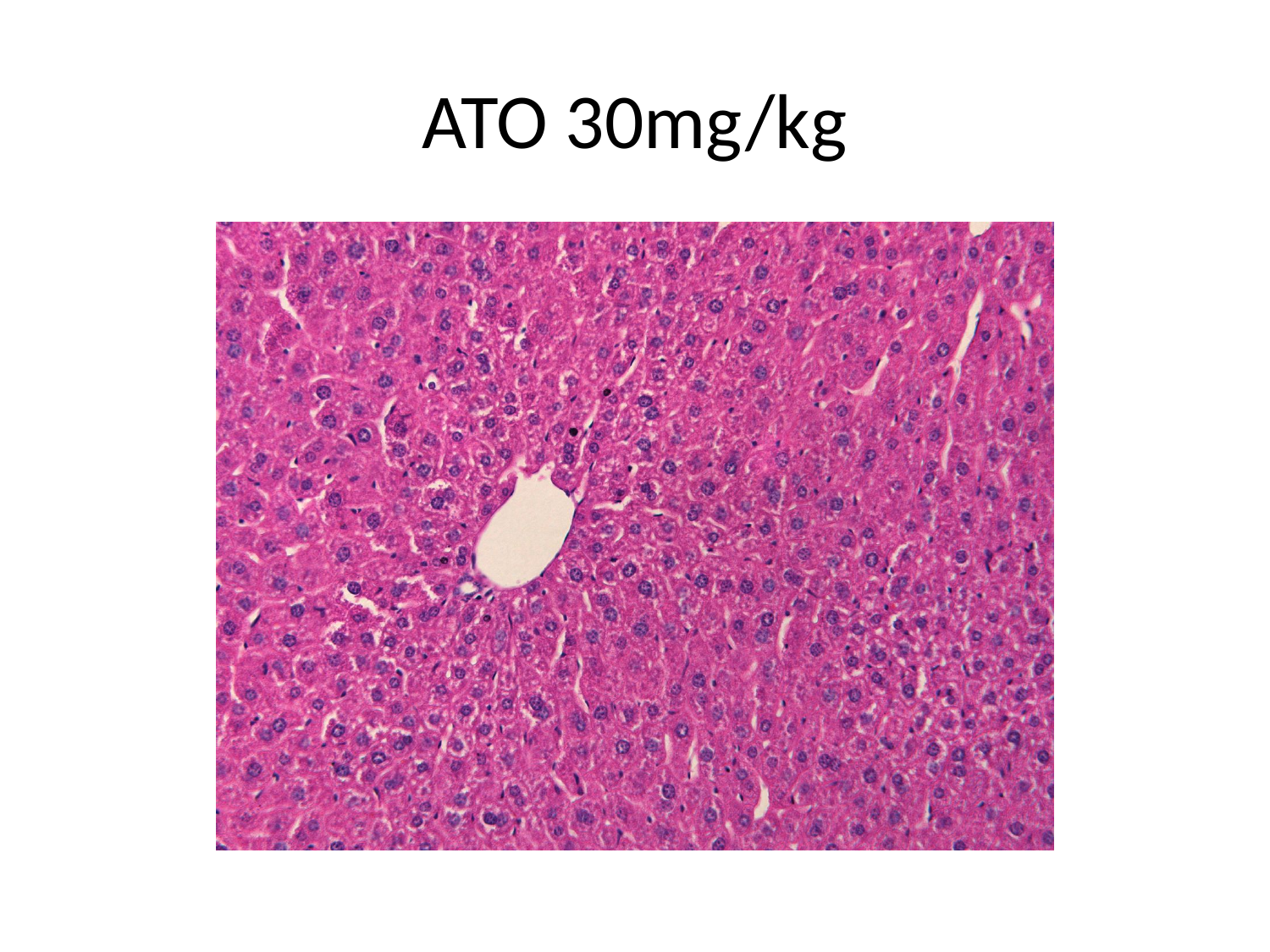

# ATO 30mg/kg

## Slide 5
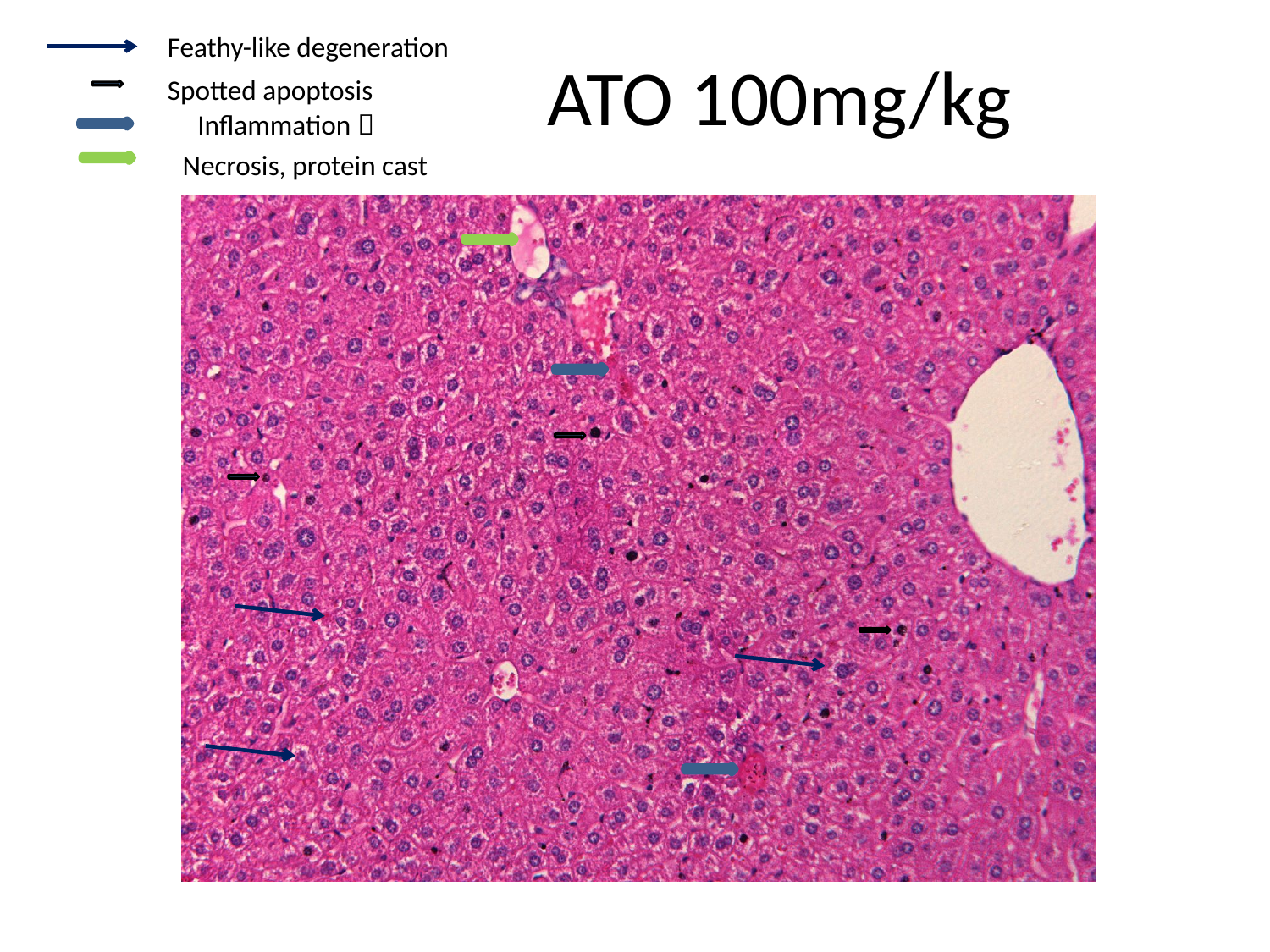

Feathy-like degeneration
# ATO 100mg/kg
Spotted apoptosis
Inflammation？
Necrosis, protein cast
